# Thrombotic complications in 2928 patients with COVID-19 treated in intensive care: a systematic review

**DOI:** 10.1007/s11239-021-02394-7

**Published:** 2021-02-14

**Authors:** William J. Jenner, Rahim Kanji, Saeed Mirsadraee, Ying X. Gue, Susanna Price, Sanjay Prasad, Diana A. Gorog

**Affiliations:** 1 Faculty of Medicine, National Heart and Lung Institute, Imperial College, London, UK; 2 Cardiology Department, East and North Hertfordshire NHS Trust, Stevenage, Hertfordshire UK; 3 Cardiology Department, Royal Brompton and Harefield NHS Foundation Trust, London, UK; 4 School of Life and Medical Sciences, Postgraduate Medical School, University of Hertfordshire, Hatfield, UK

**Keywords:** Coronavirus, Thromboembolism, Embolism, Thrombosis, Systematic review, Critical care

## Abstract

A prothrombotic state is reported with severe COVID-19 infection, which can manifest in venous and arterial thrombotic events. Coagulopathy is reflective of more severe disease and anticoagulant thromboprophylaxis is recommended in hospitalized patients. However, the prevalence of thrombosis on the intensive care unit (ICU) remains unclear, including whether this is sufficiently addressed by conventional anticoagulant thromboprophylaxis. We aimed to identify the rate of thrombotic complications in ICU-treated patients with COVID-19, to inform recommendations for diagnosis and management. A systematic review was conducted to assess the incidence of thrombotic complications in ICU-treated patients with COVID-19. Observational studies and registries reporting thrombotic complications in ICU-treated patients were included. Information extracted included patient demographics, use of thromboprophylaxis or anticoagulation, method of identifying thrombotic complications, and reported patient outcomes. In 28 studies including 2928 patients, thrombotic complications occurred in 34% of ICU-managed patients, with deep venous thrombosis reported in 16.1% and pulmonary embolism in 12.6% of patients, despite anticoagulant thromboprophylaxis, and were associated with high mortality. Studies adopting systematic screening for venous thrombosis with Duplex ultrasound reported a significantly higher incidence of venous thrombosis compared to those relying on clinical suspicion (56.3% vs. 11.0%, p < 0.001). Despite thromboprophylaxis, there is a very high incidence of thrombotic complications in patients with COVID-19 on the ICU. Systematic screening identifies many thrombotic complications that would be missed by relying on clinical suspicion and should be employed, with consideration given to increased dose anticoagulant thromboprophylaxis, whilst awaiting results of prospective trials of anticoagulation in this cohort.

**Supplementary Information:**

The online version contains supplementary material available at 10.1007/s11239-021-02394-7.

## Highlights

- The risk of thrombotic complications increases with the severity of COVID-19.
- Thromboprophylaxis is recommended for hospitalised patients, but the effectiveness of this, and the incidence of thrombotic events in patients managed in the intensive care unit (ICU) is unknown.
- In 28 studies assessing 2928 critically-ill patients with COVID-19 on the ICU, the incidence of thrombotic events was 34%, but studies employing systematic screening reported a significantly higher incidence of venous thrombosis compared to those relying on clinical suspicion alone (56.3% vs. 11.0%, p < 0.001), despite anticoagulant thromboprophylaxis.
- Consideration should be given to systematic screening and increased dose anticoagulant thromboprophylaxis in patients with COVID-19 on the ICU.

## Introduction

A prothrombotic state, attributable to a cytokine storm induced by severe acute respiratory syndrome coronavirus 2 (SARS-Cov-2) and leading to activation of the coagulation cascade, is a recognised feature of severe Coronavirus disease 2019 (COVID-19) infection. This can manifest in venous thromboembolism (VTE), arterial thrombosis and disseminated intravenous coagulation (DIC) and coagulopathy is reflective of more severe disease and adverse prognosis [1]. A significant number of patients with COVID-19 require single or multiple organ support on the Intensive Care Unit (ICU), estimated to be between 12 and 17% of patients [2–5]. with the reported mortality in these cohorts between 25 and 40% [2, 6].

Recent international guidelines recommend that hospitalized patients with COVID-19 who are immobile, have respiratory failure or co-morbidities, as well as those requiring intensive care, should receive pharmacological prophylaxis against VTE, in the absence of contraindications [7–9]. However, studies have raised concern that despite anticoagulant thromboprophylaxis, patients with COVID-19 on the ICU are at high risk of thromboembolic events [10]. Currently the exact prevalence of thrombosis in ICU-admitted patients with COVID-19 remains uncertain and in particular, whether this is sufficiently addressed by pharmacological thromboprophylaxis. Other published reviews of thrombotic complications associated with COVID-19, to date, have not specifically examined the rate of thrombotic complications in ICU-treated patients with COVID-19, nor the role of systematic screening for VTE in this cohort [11–17]. The aim of this systematic review was to identify the rate of thrombotic complications in patients with COVID-19 admitted to ICU to inform recommendations for diagnosis and management.

## Methods

The present systematic review was performed in accordance with the Cochrane Handbook for Systematic Reviews and Interventions [18], using the Preferred Reporting Items for Systematic Reviews and Meta-Analyses (PRISMA) guidelines [19], and registered in the PROSPERO database (CRD42020192147).

### Search strategy

The Pubmed/MEDLINE database was searched on 10th November 2020 for articles between 1 January 2020 and 10th November 2020 that included keywords related to COVID-19 (Wuhan coronavirus 2019, 2019-nCoV, 2019nCoV, COVID-19, SARS-CoV-2), venous thrombosis, arterial thrombosis, stroke, myocardial infarction and mesenteric ischaemia (search codes are shown in Supplementary eTable 3). Two authors (W.J. and R.K.) independently screened articles. Reference lists of included studies, relevant articles, and related systematic reviews were assessed. Eligible articles were reviewed in depth, and disagreements or queries were resolved by consensus (D.A.G., W.J., R.K.).

### Study selection

We included peer-reviewed observational studies and registries, both prospective and retrospective, which reported on thrombotic complications in patients with COVID-19 admitted to the ICU. Articles were only included if either the whole population or a subgroup of the main population were admitted to ICU, and the incidence of thrombosis in the ICU group was documented. Studies that reported on patients receiving extracorporeal membrane oxygenation (ECMO) were also included, given the specific consideration needed for thrombosis in these patients. Case reports or series, autopsy studies, articles not available in the English language, papers that repeated data already included in prior analysis and radiology studies which only selectively included those patients with radiological abnormalities, were excluded.

### Data extraction

Full text articles of eligible studies were reviewed for data extraction by two authors (W.J., R.K.). Information extracted included patient demographics (age, gender, country of admitting centre, comorbidities), requirement for renal replacement therapy (RRT) or ECMO, thromboprophylaxis or anticoagulation upon admission to ICU (how many patients, what type and dose), method of identifying thrombotic complications (clinical suspicion or routine screening), incidence and type of venous or arterial thrombotic event, dates or duration of follow-up, and reported outcomes (hospital/ICU length of stay, mortality). In studies where a subgroup of ICU patients was included, only the results from this subgroup were included in data extraction.

### Outcome measures

The primary outcome measure was the rate of occurrence of arterial or venous thrombotic complications. Secondary outcomes included the type of thrombosis including arterial or venous, hospital/ICU length of stay and mortality. Secondary analyses compared the rate of thrombotic complications between patients who were assessed with routine screening for VTE and those who were not.

### Risk of bias within individual studies

The risk of bias (low, moderate, high or critical) of the included studies was assessed using the Cochrane Collaboration tool and based on the The Risk Of Bias In Non-randomized Studies of Interventions (ROBINS-I) assessment tool [20]. Given the limited number of publications and the recent interest in COVID-19, we did not assess the risk of publication bias as it is likely that both positive and negative findings would be published early in the course of the disease, although a bias in favour in publication of positive results cannot be excluded.

### Statistical analysis

Continuous variables are presented as mean and standard deviation, or median and inter-quartile range (IQR) for normal and non-normal distributions. Dichotomous variables are presented as frequencies and percentages. The chi-squared test was used to assess differences. p < 0.05 was taken as statistically significant. All analyses were performed using IBM SPSS Statistics Version 26.

## Results

### Search results

**Fig. 1:**
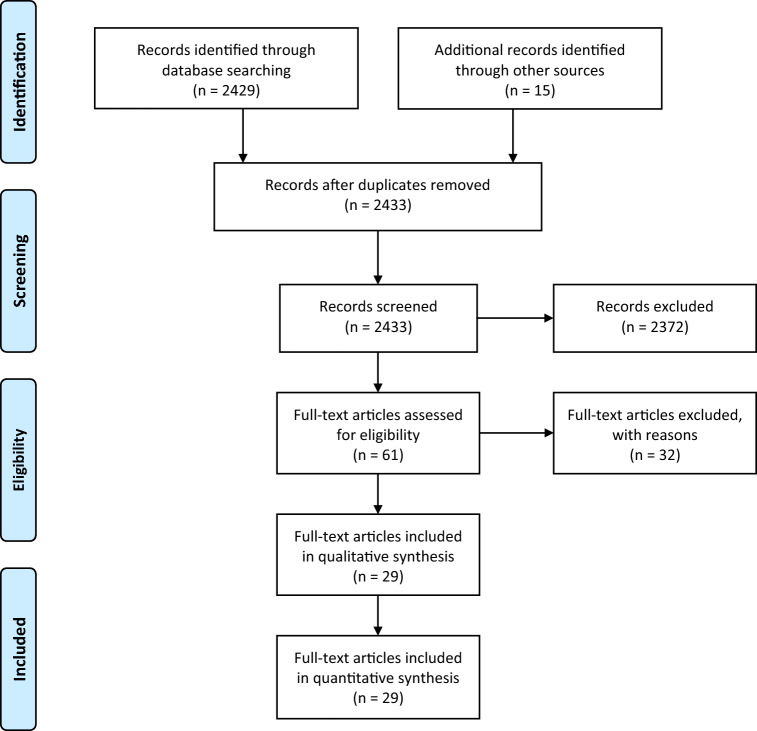
Flowchart of literature review

We identified 2429 articles on direct database search, with a further 15 records identified through other sources (Fig. 1). After de-duplication, screening article titles and abstracts, 2372 records were removed as not relevant to this review. The remaining 61 articles had assessment of the full text for eligibility. Of these, 32 were excluded; 14 due to absence of data on ICU patients, 6 because they only included a subgroup of patients who had undergone CT scans, 3 because they only included patients who had undergone ultrasound doppler, 2 because they repeated data from other studies, 2 because they were small case series, 2 that did not adequately quantify thrombotic outcomes, 2 because they only reported outcomes on patients who had a diagnosis of pulmonary embolism (PE) or stroke, and 1 because the article was not peer reviewed.

In total, 29 articles (28 studies) were included in the main analysis. Of these, 22 studies were specific to ICU patients, and 6 studies included a mixed population, with specific reporting on the subgroup of patients admitted to ICU. Two papers reported on the same patient group, with the second providing additional data to its predecessor [10, 21].

There was significant variation in the incidence of thrombotic events. Few studies reported all thrombotic event types (some only reported one type of thrombotic event); disease severity differed amongst studies with 4 studies reporting exclusively on patients on ECMO. Importantly, some studies used routine screening for thrombotic complications whilst others did not.

### Study and patient characteristics

A total of 2928 patients were included and their clinical characteristics are shown in Tables 1. and 2. Where described (23 studies), mean patient age ranged from 45 to 70 years and 69% of subjects were male (24 studies). Although not all studies documented comorbidities, roughly a third of patients had diabetes, and there was a high prevalence of obesity, hypertension and cardiovascular disease. Nine studies documented use of ECMO. Eight studies documented use of RRT, which was employed in 18% of those patients.

**Table 1:** Patient characteristics and treatments received

Patient demographics and treatments	Mean (SD), Median (IQR) or patients with characteristic/total patients with this characteristic documented or n(%)	% of total patients who have this variable documented	Number of studies reporting on this demographic (Specific references in brackets)
Age (years)	59.9(14.1), 62(56–66), 70(62–80), 63(53–71), 64(12), 68(51.5–74.5), 61(16), 59(13), 61(55–69), 62.2(8.6), 61(55–70), 65(range:32–97), 59(49–66), 64.5(11.8), 61(14), 62(53–69), 57(49–64), 61.7(15.8), 45(26–66), 68(11), 59(50–61), 50(43–62), 64(57–71)	1788/2928 (61%)	23 [10, 21–37, 39, 40, 51–53]
Male	1291/1879 (69%)	1879/2928 (64%)	24 [10, 21–33, 36, 37, 39, 40, 51–54]
Diabetes	426/1393 (31%)	1393/2928 (48%)	16 [10, 21, 22, 24, 25, 27–31, 33, 34, 36, 37, 51–54]
Obesity	146/392 (37%)	392/2928 (14%)	3 [52, 53, 55]
Body mass index (kg/m^2^)	29.5(29.3–32.4), 30.2(25.5–33.5), 34.8(11.8), 31.4(9.0), 30(26–35), 28(24–34), 30.3(5.4), 27(24–29), 28 (25–32), 28(25–32), 30.3(5.7), 27.5(4.6), 27.8(25.1–33.9), 31(27–36)	942/2928 (32%)	14 [22, 24–27, 29–31, 36, 39, 40, 52, 54, 56, 57]
Hypertension	498/1021 (49%)	1021/2928 (35%)	13 [10, 21, 22, 25, 27, 28, 30, 31, 33, 36, 37, 39, 52–54]
Cardiovascular disease	281/1393 (20%)	1393/2928 (48%)	15 [10, 21, 22, 25, 27–29, 31, 33, 36–38, 51–54, 58]
Cerebrovascular disease	39/708 (6%)	708/2928 (24%)	6 [28, 36, 38, 52–54]
Atrial fibrillation	21/287 (7%)	287/2928 (10%)	3 [22, 24, 36]
Renal replacement therapy	147/834 (18%)	834/2928 (28%)	8 [10, 21, 26, 37, 38, 54–57]
ECMO	67/529 (13%)	529/2928 (18%)	9 [23, 25, 29, 36, 38–40, 57, 59]

*ECMO* extracorporeal membrane oxygenation, *IQR* inter-quartile range, *SD* standard deviation

**Table 2:** Incidence of thrombotic complications in the whole cohort and in patients with and without routine ultrasound screening for deep venous thrombosis

Type of thrombosis	Incidence of thrombotic complication in total patient cohort (n = 2929)	Incidence of event in patients not routinely screened for DVT	Incidence of event in patients routinely screened for DVT	Comparison of the incidence of thrombosis with screening vs. without screening
DVT	431/2671 (16.1%)	256/2328 (11.0%)	151/268 (56.3%)	56.3% vs. 11.0% **(p < 0.001)**
PE	325/2580 (12.6%)	277/2298 (12.1%)	37/207 (17.9%)	17.9% vs. 12.6% **(p = 0.009)**
CVA	52/1736 (3.0%)	52/1736 (3.0%)	NA	NA
MI	137/1736 (8.0%)	137/1736 (8.0%)	NA	NA
Line thrombosis	13/574 (2.3%)	13/574 (2.3%)	NA	NA
Limb or Mesenteric ischaemia	39/1566 (2.5%)	39/1566 (2.5%)	NA	NA
Oxygenator thrombosis (ECMO)	13/48 (27.1%)	13/48 (27.1%)	NA	NA
Circuit thrombosis (RRT)	28/29 (96.6%)	28/29 (96.6%)	NA	NA

*CVA* cerebrovascular accident, *DVT* deep venous thrombosis, *ECMO* extracorporeal membrane oxygenation, *MI* myocardial infarction, *NA* not available, *PE* pulmonary embolism, *RRT* renal replacement therapy

### Baseline VTE prophylaxis and anticoagulation

Twenty-four studies documented whether venous thromboprophylaxis or anticoagulation was used. Twenty-one studies described the use of anticoagulation, 20 studies documented prophylactic anticoagulation, and 8 defined the anticoagulant dose used. There was significant variability between studies on the type, dose and indication for prophylactic or therapeutic anticoagulation, and within some studies the thromboprophylaxis dosing policy changed during the analysis (Table 3).

**Table 3:** Characteristics of the studies analysed, including patient cohorts, anticoagulant thromboprophylaxis and thrombotic complications

Study	Total no. patients	Age mean (SD) or median (IQR)	Gender	Diabetes n (%)	BMI mean (SD) or median (IQR)	Patients on ECMO n (%)	Patients on thromboprophylaxis on admission n (%)	Type of thromboprophylaxis	Patients on therapeutic anticoagulation n (%)	Routine screening for VTE	Patients with any venous thrombotic complications n (%)	Patients with PE n (%)	Patients with DVT n (%)	Other thrombotic complicationsn (%)	Patients with arterial thrombotic events (%)	Patients with any reported thrombotic events n (%)
Piazza et al. [36]	170	61.7 (15.8)	M 106 (62%)	66/170 (39)	30.3 (5.7)	NA	89%	LMWH UFH Rivaroxaban	NA	No	46/170 (27)	3/170 (2)	39/170 (23)	Catheter/device-related thrombosis: 30/39 (77) Catheter/device-related arterial thrombosis: 11/170 (7)	14/170 (8)	60 (35)
Longhitano et al. [32]	62	NA	NA	NA	NA	NA	100%	Enoxaparin 100U/kg/24 h > 80 kg: Enoxaparin 5000U b.i.d	0 (0)	No	12/62 (19)	11/62 (18)	4/62 (6)	NA	NA	12/62 (19)
Shah et al. [52]	187	57 (49–64)	M 124 (66%)	54/187 (30)	28 (25–32)	5 (3)	151/187 (81)	LMWH UFH	27/187 (14)	No	56/187 (30)	42/187 (23%)	22/187 (12)	Extracorporeal circuit disruption: 23 (12)	25/187 (13)	81/187 (43%)
Bilaloglu et al. [41]	829	NA	NA	NA	NA	NA	‘Most received thromboprophylaxis’	NA	NA	No	113/829 (14)	52/829 (6)	78/829 (9)	Limb ischaemia, renal and splenic infarcts, PVT: 18/829 (2)	154/829 (19)	224/829 (29)
Bemtgen et al. [40]	11	59 (50–61)	M 7 (64%)	NA	27.8 (25.1–33.9)	11 (100)	UFH on ECMO	UFH on ECMO (argatroban if HIT)	11/11	No	NA	NA	NA	Extracorporeal circuit disruption: 7/11 (64)	NA	7/11 (64)
Yuriditsky et al. [51]	64	64 (57–71)	M 46 (72%)	NA	NA	NA	9/64 (14)	Enoxaparin UFH	55/64 (86)	No	20/64 (31)	1/64 (2)	19/64 (30)	Clot in transit in RV: 1/64 (2)	NA	20/64 (31)
Hekimian et al. [35]	51	NA	NA	NA	NA	NA	NA	NA	NA	No	8/51 (16)	8/51 (16)	NA	NA	NA	8/51 (16)
Cui et al. [33]	81	59.9 (14.1)	M 37 (46%)	8/81 (10)	NA	NA	NA	NA	NA	No	20/81 (25)	NA	20/81 (25)		NA	20/81 (25)
Helms et al. [38]	150	63 (53–71)	M 122 (81%)	30/150 (20)	NA	12/150 (8)	105/150 (70)	LMWH: 4,000 IU/day UFH: 5–8 U/kg/h	45/150 (30)	No	59/150 (39)	25/150 (17)	3/150 (2)	Circuit thrombosis (RRT): 28/29 (97); ECMO pump thrombosis: 3/12 (25)	4/150 (3)	63/150 (42)
Klok et al. [10, 21]	184	64 (12)	M 139 (76%)	NA	NA	NA	167/184 (91) (all others receiving full anticoagulation)	Nadroparin (dose dependent on patient weight and recruiting site)	17/184 (9)	No	68/184 (37)	65/184 (35)	1/184 (0.5)	Catheter related thrombosis: 2/184 (1)	7/184 (4)	75/184 (41)
Poissy et al. [34]	107	NA	NA	NA	NA	NA	NA	LMWH or UFH	NA	No	24/107 (22)	22/107 (21)	5/107 (5)	NA	NA	24/107 (22)
Thomas et al.*.*[37]	63	59 (13)	M 44(70%)	NA	NA	NA	63/63 (100)	Dalteparin	NA	No	6/63 (10)	5/63 (8)	0/63 (0)	Line thrombosis: 1/51 (2)	2/63 (3)	8/63 (13)
Lodigiani et al. [53]	61	61 (55–69)	M 49 (80%)	11/61 (18)	Obesity: 28%	NA	59/61 (97)	LMWH	2/61 (3)	No	4/61 (7)	2/61 (3)	1/61 (2)	Line thrombosis:1/61 (2)	4/61 (7)	8/61 (13)
Beyls et al. [39]	12	62 (56–66)	M 10 (83%)	NA	29.5 (29.3 – 32.4)	12/12 (100)	UFH on ECMO	UFH on ECMO	12/12 (100)	No	10/12 (83)	1/12 (8)	6/12 (50)	Cannula thrombosis 2/12 (17); ECMO Oxygenator thrombosis 1/12 (8)	NA	10/12 (83)
Maatman et al. [56]	109	61 (16)	M 62 (57%)	43/109 (39)	34.8 (11.8)	NA	102/109 (94)	UFH: 5,000 IU S/C t.d.s. Enoxaparin: 40 mg S/C o.d. or 30 mg S/C b.i.d	7/109 (6)	No	31/109 (28)	5/109 (5)	29/109 (27)	Line thrombosis:5/109 (5)	NA	31/109 (28)
Fraissé et al. [54]	92	61 (55–70)	M 73 (79%)	35/92 (38)	30 (26–35)	NA	43/92 (47)	LMWH	49/92 (53)	No	31/92 (34)	25/92 (27)	12/92 (13)	NA	8/92 (9)	37/92 (42)
Al Samkari et al. [55]	144	65 (32–97)	M 93 (65%)	58/144 (40)	Obesity 62 (43%)	NA	124/144 (86)	Unknown	18/144 (13)	No	11/144 (8)	NA	NA	Line thrombosis 2/144 (1); Circuit thrombosis (RRT): 8/12 (67)	8/144 (6)	26/144 (18)
Hippensteel et al. [59]	91	NA	M 53 (58%)	28/91 (31)	NA	0/91 (0)	NA	Unknown	At start: none; later 24 anticoagulated for AF (n = 4) or unconfirmed VTE/hypercoagulability (n = 20)	No	24/91 (26)	5/91 (6)	11/91 (12)	Internal jugular vein thrombus 8 (9)	NA	24/91 (26)
Desborough et al. [57]	66	59 (49–66)	M 48 (73%)	27/66 (41)	28 (24–34)	8/66 (27)	55/66 (83)	Dalteparin (adjusted to weight and renal function)	11/66 (17)	No	10 / 66 (15)	5/66 (8)	6/66 (9)	NA	NA	10/66 (15)
Middeldorp et al. [30]	75	62 (10)	M 58 (77%)	NA	27 (24–29)	NA	68/75 (91)	Nadroparin with weight adjustment. Dose increased mid-study for ICU patients	7 (9.3)	Screening in 38/ 75 (51%) patients	35/75 (47)	11/75 (15)	24/75 (32)	NA	NA	35/75 (47)
Parzy et al. [24]	13	50 (43–62)	M 9 (70%)	2/13 (15)	31 (27–36)	13/13 (100)	UFH on ECMO	UFH on ECMO (argatroban if HIT)	11/11 (100)	Yes (Contrast CT)	13/13 (100)	3/13 (23)	11/13 (85)	Oxygenator thrombosis: 1/13 (8) centrifugal pump thrombosis: 1/13 (8)	NA	13/13 (100)
Mak et al. [23]	51	45 (26–66)	M 38 (75%)	NA	NA	51 (100)	NA	NA	NA	Yes (CTPA)	NA	18/51 (35)	NA	NA	NA	18/51 (35)
Longchamp et al. [22]	25	68 (11)	16 (64%)	1 (4)	27.5 (4.6)	NA	23/25 (92)	Enoxaparin (according to weight) UFH	2/25 (8)	Yes	8/25 (32)	5/25 (20)	6/25 (24)	NA	NA	8/25 (32)
Voicu et al. [27]	92	62 (53–69)	M 66 (72%)	30 (33)	28 (25–32)	NA	68 (74)	LMWH UFH	24 (26)	Yes	48/92 (52)	5/92 (5)	44/92 (48)	NA	NA	48/92 (52)
Criel et al. [29]	30	64.5 (11.8)	M 20 (67%)	5/30 (17)	30.3 (5.4)	0/30 (0)	30/30	Exoxaparin 40 mg × 2 (or 60 mg × 2 if weight > 100 kg)	0/30 (0)	Yes	4/30 (13)	NA	4/30 (13)	NA	NA	4/30 (13)
Llitjos et al. [26]	26	68 (51.5–74.5)	M 20 (77%)	NA	30.2 (25.5 – 33.5)	2/26 (8)	8/26 (31)	LMWH	18/26 (69)	Yes	24/26 (92)	6/26 (23)	18/26 (69)	NA	NA	24/26 (92)
Ren et al. [28]	48	70 (62–80)	M 26 (54%)	13/48 (27)	NA	NA	47/48 (98)	LMWH	0/48 (0)	Yes	41/48 (85)	NA	41/48 (85)	NA	NA	41/48 (85)
Nahum et al. [25]	34	62.2 (8.6)	M 25 (74%)	15/34 (44)	31.4 (9.0)	4/34 (12)	34/34 (100)	Unknown	0/34 (0)	Yes	27/34 (79)	NA	27/34 (79)	NA	NA	27/34 (79)

*AF* atrial fibrillation, *BMI* body mass index, *b.i.d.* = bis in die (twice daily), *DVT* deep venous thrombosis, *ECMO* extracorporeal membrane oxygenation, *IQR* interquartile range, *i.v.i.* intravenous infusion, *LMWH* = low molecular weight heparin, *M* male, *NA* not available, *o.d.* once daily, *PE* pulmonary embolism, *RRT* renal replacement therapy, *S/C* subcutaneous, *SD* standard deviation, *t.d.s.* ter in die (three times daily), *UFH* unfractionated heparin, *VTE* venous thromboembolism

### Identification of thrombotic complications

Twenty-four studies described the method of identifying thrombotic complications. Of these, 8 studies involved systematic screening for VTE in all patients [22–29], one study performed selective VTE screening [30], whilst 20 studies did not utilise screening and relied on clinical suspicion to undertake tests for VTE. Two ECMO studies performed a thoraco-abdominopelvic CT on all new retrievals [23, 24].

The method of screening for deep venous thrombosis (DVT) included Duplex ultrasound of the limbs upon admission to ICU [22, 25, 26, 28, 29]. In 3 studies, a repeat ultrasound was performed in patients without DVT on the first ultrasound, between 2 and 7 days after the initial scan [25, 26, 31].

### Occurrence of thrombotic complications

**Fig. 2:**
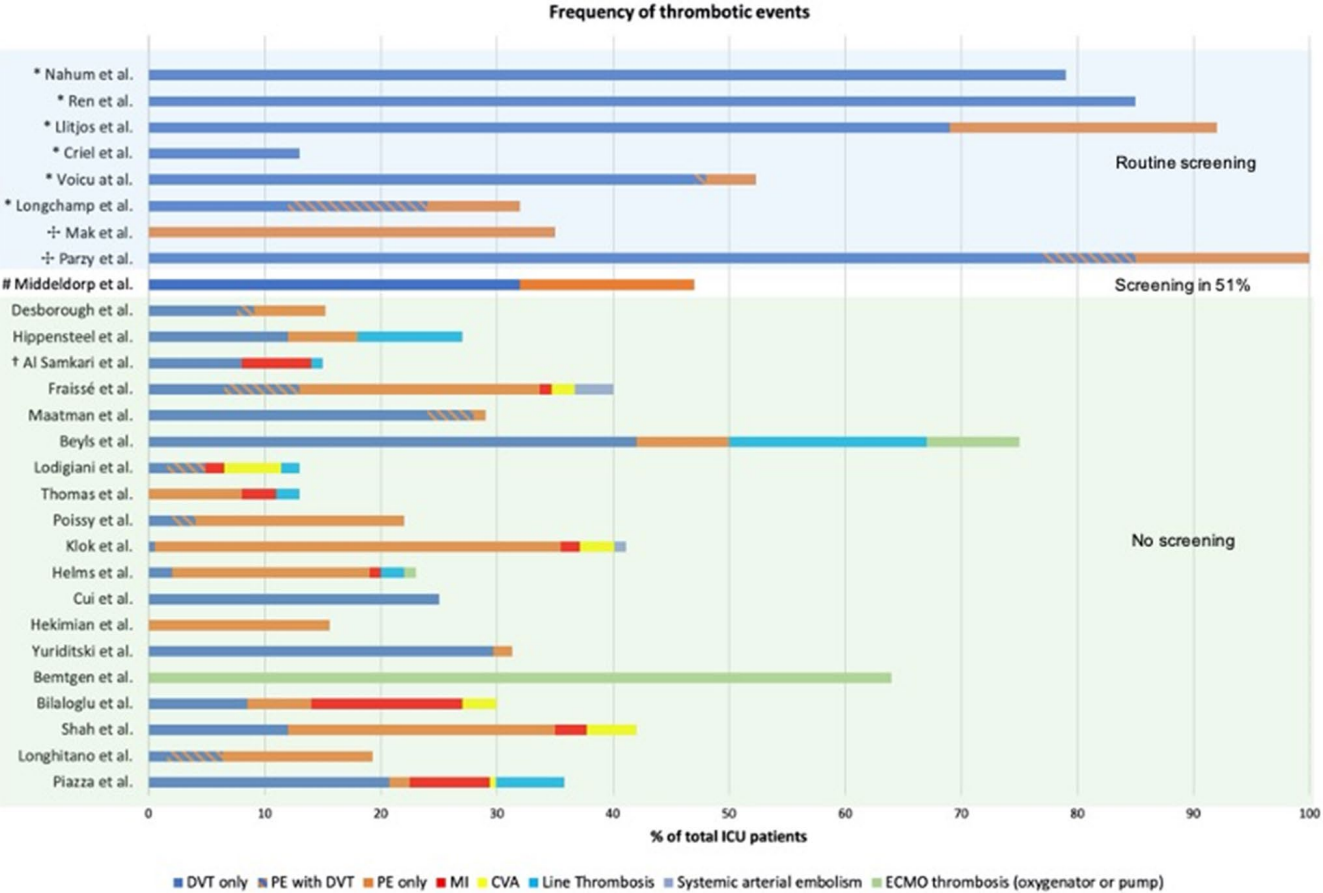
Bar chart presenting percentage of patients with thrombotic complications by study. Panel background shows studies that employed routine screening for venous thrombosis (blue) and those that relied on clinical suspicion to investigate thrombotic complications (green). * Denotes those studies employing routine screening for venous thromboses. ✣ Denotes those studies employing routine screening for venous thromboses with computed tomography. # This study employed routine screening for venous thrombosis in only 51% of patients. † This study did not differentiate the types of arterial or venous thrombotic events, although reporting 7.6% venous thrombotic events (blue) and 5.6% arterial events (red). *CVA* cerebrovascular accident, *DVT *deep vein thrombosis, *ECMO* extracorporeal membrane oxygenator, *MI* myocardial infarction, *PE* pulmonary embolism

All studies quantified at least one type of thrombotic complication (Fig. 2 and Table 2). Thrombosis of any kind was reported in 996 patients (34%). Incidence of DVT was 16.1% (reported in 24 studies) and of PE 12.6% (22 studies). The incidence of arterial thrombotic events was 12% (9 studies), which included myocardial infarction (8%), ischaemic cerebrovascular accident (3%), limb or mesenteric infarction (2.5%).

All studies concluded that the rate of thrombosis was high, and management of these patients ought to be specifically tailored to reduce thrombotic complications. Eleven studies suggested better risk assessment for thrombosis, with the use of D-dimer and improved diagnostic strategies [10, 21, 26–28, 30, 32–36], whilst 7 suggested increasing the dose of anticoagulation administered, as thrombotic events were high despite pharmacological thromboprophylaxis [10, 25, 26, 32, 34, 35, 37]. Some papers recommended routine systematic anticoagulation for all [25], while one group recommended a higher dose of thromboprophylaxis [37], which had already been adopted at that centre.

### Routine screening vs. clinical suspicion to investigate thrombosis

Studies employing routine screening reported a much higher rate of thrombotic complications than studies without routine screening (Tables 2 and 3 and Fig. 2). The pooled incidence of DVT was 11% in studies without systematic screening, whereas studies employing routine screening reported a rate of 56.3% (p < 0.001). The incidence of PE ranged from 3 to 35%. With the exception of two studies which only reported on patients on ECMO, none of the studies adopted routine screening for PE with CT pulmonary angiography.

### Thrombotic complications of patients receiving ECMO

Five studies described thrombotic events in patients receiving ECMO [23, 24, 38–40]. In the one study which performed screening, all 13 patients developed thromboembolism during ECMO, with 85% developing DVT, 23% developing PE, one a thrombotic occlusion of the centrifugal pump and one oxygenator thrombosis [24]. Amongst 51 ECMO patients where screening CT was performed, the incidence of PE was 35% [23], whilst another study reported ECMO-related thrombosis in 64% of patients, a significantly higher prevalence than that in non-COVID-19 controls [40]. Another study reported a 33% rate of thrombotic complications, including 17% mortality related to thrombotic complications [39].

### Thrombotic complications and in-hospital mortality

Of the 28 studies, 20 reported in-hospital mortality, ranging from 9 to 54% (eTable 1). Nine studies reported on the differential mortality between those with and without thrombotic complications. One cohort study of 184 patients showed that patients with VTE had higher mortality than patients without thromboembolism [10, 21]. However, a subsequent larger study of 829 ICU patients showed similar mortality rate between those who developed any thrombotic event (59.8%) and those who did not (52.1%) [41]. At the point of analysis, 14 studies reported that patients remained in hospital, ranging from 6 to 83% of patients. Eight studies reported the number of patients who had been discharged from hospital, ranging from 0 to 79% of patients. The length of hospital stay and duration of follow up were only documented in a few studies (eTable 1).

### Risk of bias within individual studies

The risk of bias in the included studies was assessed using the Cochrane Collaboration tool (eTable 4). Thirteen studies showed a high risk of bias, notably selection bias secondary to early reporting of results, without a set follow-up period. There is also significant reporting bias, due to limited reporting of all thromboembolic complications.

## Discussion

This review demonstrates a very high incidence of thrombotic complications in patients with COVID-19 admitted to ICU, despite anticoagulant thromboprophylaxis. Secondly, it is clear that thrombotic complications are frequently undetected in the ICU setting, as evidenced by the very high incidence in studies that employed systematic screening for thrombotic complications, compared to those that relied on clinical suspicion to trigger investigation.

The rate of thrombotic complications appears significantly higher than that seen in patients with non-COVID-19 sepsis or pneumonia admitted to ICU. One of the studies included in our review compared COVID-19 patients with matched non-COVID-19 patients with ARDS on the ICU and showed a much higher rate of thrombotic complication with COVID-19 (11.7% vs. 4.8%) [38]. Similarly, Poissy et al. reported a much higher rate of thrombotic complications in patients admitted to ICU with COVID-19 than in ICU-admitted patients with influenza (20.6% vs 6.1%) [34]. A report on 198 hospitalised patients with COVID-19 receiving thromboprophylaxis (75 of whom were treated on ICU), showed the incidence of thrombotic complications increased over time and was related to increased mortality [30].

Clinically, thrombotic complications are often difficult to recognise in intubated patients, particularly in patients with COVID-19, where any deterioration in lung function due to PE or pulmonary thrombosis may be assumed to be part of the clinical progression of the ARDS. Furthermore, CT imaging may be less frequently performed due to the challenges and risks associated with moving critically unwell ventilated patients to a scanner, complicated further by the necessity to limit intra-hospital COVID-19 transmission. However, the high incidence of thrombotic complications in studies using systematic screening implies that clinical suspicion alone results in significant under-detection of thromboembolic events on ICU. This is supported by autopsy studies which show high rates of thrombosis at the macrovascular and microvascular level [42].

A prothrombotic state is an emerging hallmark of severe COVID-19 and elevations in D-dimer and prothrombin time are well documented and related to increased mortality [43], with severe coagulation abnormalities reported in almost all patients with severe disease [44, 45]. However, measurement of D-dimer level is generally not helpful in predicting thrombotic complications in ICU-treated patients, in particular given the significant baseline elevations in this cohort [46], supporting the case for systematic imaging in this cohort.

The importance of anticoagulant thromboprophylaxis in patients hospitalised with COVID-19 is well recognised. In March, the International Society on Thrombosis and Haemostasis (ISTH) and the American Society of Haematology recommended that all hospitalized COVID-19 patients should receive prophylactic-dose low molecular weight heparin (LMWH) unless contraindicated [47, 48]. The American College of Chest Physicians (CHEST) expert panel guideline, published on June 2, 2020 [7] recommends standard dose anticoagulant thromboprophylaxis in ICU patients, and does not advocate addition of mechanical prophylaxis (i.e. intermittent pneumatic compression) to pharmacological thromboprophylaxis. On the other hand, the latest ISTH consensus statement published on May 27, 2020 [8] whilst recommending routine thromboprophylaxis in COVID-19 patients on the ICU with preferably standard-dose LMWH or unfractionated heparin, recommended that patients with obesity should be considered for a 50% increase in the dose of thromboprophylaxis and multi-modal thromboprophylaxis with mechanical methods should be considered. Furthermore, intermediate-dose LMWH for ICU patients was advocated by up to 50% of ISTH respondents. This is supported by an analysis of 2,733 hospitalised patients with COVID-19 in New York which reported that anticoagulation improved survival, and subgroup analysis indicated that use of treatment-dose anticoagulation (received by 28% of patients) may be associated with improved survival compared to no-anticoagulation or prophylactic-dose anticoagulation, particularly in patients receiving mechanical ventilation [49]. A retrospective evaluation of 3480 patients with COVID-19 of whom 18% required ICU, showed the benefit of anticoagulation in reducing mortality appeared to be dose-dependent, with the greatest impact in those with critical illness [50].

The current anticoagulant thromboprophylaxis employed by the majority of studies reported here appears insufficient in patients with COVID-19 managed on the ICU. There may be a number of possible explanations for this. Firstly, the prothrombotic state in severe COVID-19 sepsis may be much more profound than previously appreciated, and more severe than in patients with severe sepsis of other aetiology. Further, owing to viral adhesion occuring on the ACE2 receptor on endothelial cells, the degree of endothelial dysfunction through viral replication, inflammatory cell infiltration and apoptosis may be greater [46]. Secondly, because of the difficulty in detecting VTE on the ICU, it is possible that in some patients thrombotic complications developed before admission to ICU and perhaps even before thromboprophylaxis was instituted. Thirdly, obesity is highly prevalent amongst this cohort and it is possible that the dose of thromboprophylaxis may have been insufficient for those with extreme BMI.

This review also highlights the high incidence of arterial thrombotic events, corroborated by other studies [60, 61]. In the setting of COVID-19, myocardial injury, defined by an increased troponin level, is predominantly attributable to non-ischaemic myocardial processes. Thus, whilst many early reports in patients with COVID-19 equated a troponin rise with myocardial infarction, typically this is more like to be related to sepsis and associated systemic inflammatory response, pro-coagulant status, and myocarditis. Without regular screening with cardiac biomarkers, in the absence of major ECG changes, myocardial injury in patients with COVID-19 can be frequently missed and yet is associated with an increased mortality [62, 63]. Likewise, stroke can be very challenging to recognise in patients who are intubated and ventilated, due to masking of clinical signs and symptoms with sedation and neuromuscular blockade. Unlike the case with the use of anticoagulation for thromboprophylaxis of venous thrombotic events, there are no convincing data that use of antiplatelet agents (or indeed anticoagulation) can reduce the frequency of arterial thrombotic events in patients with COVID-19 on the ICU.

### Limitations

There is inherent bias in all observational cohort studies. Selection bias may favour the identification and publication of case cohorts with high rates of thrombotic complications. The retrospective nature of many studies will add bias. Differences between studies will add to bias, including variation in the ICU population studied (some including the highest risk patients on ECMO and RRT, others including lower risk patients requiring non-invasive ventilation), differential methods of diagnosing COVID-19 (whether by nasopharyngeal swab or antibody testing), variation in type and dose of anticoagulant thromboprophylaxis and differential thresholds for investigating thrombotic complications. Some papers report only one type of thrombosis, whilst others report all available thrombosis data. Furthermore, differences in follow-up between studies will underestimate rates of thromboses, with varilable proportions of the study population remaining in hospital, on ICU, or even on ECMO at the point of publishing [39]. We compared studies that used systematic screening to those depending on clinical suspicion to diagnose thrombotic complications. In addition, differences in patient cohorts, both in terms of characteristics reported (such as age, obesity, diabetes, country/type of healthcare where study was undertaken) and those not reported, such as other co-morbidities, medications and ethnicity, the latter in particular is highly pertinent as black, Asian and minority ethnic (BAME) groups have been reported to be adversely affected by COVID-19.

## Conclusion

Despite anticoagulant thromboprophylaxis, there is a very high incidence of thrombotic complications in patients with COVID-19 admitted to ICU, and systematic screening identifies many thrombotic complications that would be missed by relying on clinical suspicion to trigger investigation. Systematic screening for VTE is therefore recommended in this cohort, and higher dose thromboprophylaxis should be considered, whilst awaiting the results of prospective studies to guide anticoagulation in patients with severe COVID-19 disease.

## Supplementary Information

Below is the link to the electronic supplementary material.

Supplementary file1 (DOC 199 KB)

## Abbreviations

BMI: Body mass index
COVID-19: Corona virus disease 2019
DIC: Disseminated intravascular coagulation
DVT: Deep venous thrombosis
ECMO: Extra-corporeal membrane oxygenation
ICU: Intensive care unit
LMWH: Low molecular weight heparin
PE: Pulmonary embolism
RRT: Renal replacement therapy
SARS-Cov-2: Severe acute respiratory syndrome coronavirus 2
VTE: Venous thromboembolism
