# Supplementary material for: Thrombotic complications in 2928 patients with COVID-19 treated in intensive care: a systematic review
Source: J Thromb Thrombolysis. 2021 Feb 14;51(3):595–607. doi: 10.1007/s11239-021-02394-7 (PMC7882250; doi:10.1007/s11239-021-02394-7)
Supplement: Supplementary file 1 — Supplementary file1 (DOC 199 KB) [file 11239_2021_2394_MOESM1_ESM.doc]

eTable 1.

| **Study** | **Dates of follow-up, or duration in days (median, IQR unless stated otherwise)** | **Hospital length of stay [LOS] (median, IQR unless stated otherwise)** | **Discharged from hospital at time of analysis,**  **n (%)** | **Remain hospitalised at time of analysis,**  **n (%)** | **Remain on ICU at time of analysis,**  **n (%)** | **Overall mortality rate at time of analysis, n (%)** | **Patients with all reported thrombotic events,**  **n (%)** | **Mortality Rate in those with reported VTE,**  **n (%)** | **Mortality rate in those without reported VTE,**  **n (%)** |
| --- | --- | --- | --- | --- | --- | --- | --- | --- | --- |
| Cui *et al.* | 30/1/20-22/3/20 | NA | 64/81 (79) | 9/81 (11) | NA | 8/81 (10) | 20/81 (25) | 8/20 (40) | 0/61 (0) |
| Helms *et al.* | At least 7 days | ICU LOS 9.4 ± 4.2 | NA | *36/150 (24) had been discharged from ICU* | 101 (67) | 13/150 (9) | 63/150 (42) | NA | NA |
| Klok *et al.* | 14 (6-19) | NA | 78/184 (43) | 65/184 (35) | NA | 41/184 (22) | 75/184 (41) | Patients with VTE had a higher risk of death: HR 5.4 (95%CI 2.4–12) | |
| Poissy *et al.* | NA | ICU LOS 15 [range, 10-30] | 48/107 (45) | 44/107 (41) | NA | 15/107 (14) | 27/107 (25) | NA | NA |
| Thomas *et al.* | 8 (range 1-28) | NA | NA | 20/63 (32) *had been discharged from ICU* | 33/63 (52) *on ICU or transferred to ECMO centre* | 10/63 (16) | 8/63 (13) | NA | NA |
| Lodigiani *et al.** | 13/2/20 – 10/4/20 | 18 (14-24) | NA | NA | NA | NA | 8/61 (13) | 0/8 (0) | NA |
| Beyls *et al.* | 1/3/20-4/4/20 | NA | 0/12 (0) | 10/12 (83) | 10/12 (83) | 2/12 (17) | 10/12 (83) | 2/10 (20) | 0/2 (0) |
| Maatman *et al.* | 12/3/20-6/5/20 | 20 (16-27) *in patients discharged* | 75/109 (69) | 7/109 (6) | 3/109 (3) | 27/109 (25) | 39/109 (36) | 8/31 (26) | 19/78 (24) |
| Llitjos *et al.* | 19/3/20-11/4/20 | NA | NA | 16/26 (62) *discharged from ICU* | 7/26 (27) | 3/26 (12) | 24/26 (92) | NA | NA |
| Ren *et al.* | 29/2/20-2/3/20 | NA | NA | NA | NA | 15/48 (31) | 41/48 (85) | 13/41 (32) | 2/7 (29) |
| Nahum *et al.* | 3/20 – 4/20 | NA | NA | NA | NA | NA | 27/34 (79) | NA | NA |
| Fraisse *et al.* | 6/3/20-6/5/20 | NA | NA | 28/92 (30) *discharged from ICU* | 25/92 (27) | 38/92 (41) | 39/92 (42) | 18/37 (49) | 20/55 (36) |
| Al Samkari *et al.* | 1/3/20-5/4/20, 461 patient-weeks | 9 (range 2-23) *where hospitalisation complete* | 18/144 (13) | 99/144 (69 | NA | 27/144 (19) | 26/144 (18) | NA | NA |
| Hippensteel *at al.* | 18/3/20-6/5/20 | 26(7) in VTE group, 16 (10) in no-VTE group, mean (SD) | 44/91 (48) | 25/91(27) | NA | 22/91 (24) | 24/91 (26) | 2/24 (9) | 20/67 (30) |
| Desborough *et al.* | 1/3/20-31/3/20, follow up 28 days after ICU admission or until death / last seen alive | 15 (9-24) | 38/66 (58) | 8/66 (12) | NA | 20/66 (30) | 10/66 (15) | NA | NA |
| Criel *et al.* | 3/4/20-22/4/20 | NA | NA | NA | NA | 4/30 (13) | 4/30 (13) | NA | NA |
| Middeldorp *et al.** | 2/3/20-30/4/20 | 15 (9-20) | NA | NA | NA | NA | 35/75 (47) | NA | NA |
| Voicu *et al.* | 3/11/19-5/5/20 | NA | NA | NA | NA | NA | 48/92 (52) | NA | NA |
| Longhitano *et al.** | 1/3/20-31/3/20 | NA | NA | NA | NA | NA | 12/62 (19) | NA | NA |
| Shah *et al.* | 15/3/20-15/5/20 | 15 (7-21) | NA | 95/187 (51) *discharged from ICU* | 33/187 (18) | 59/187 (32) | 81/187 (43) | 32/81 (40) | 27/106 (26) |
| Piazza *et al.* | 30 days | 16 (8-24) | NA | 50/170 (29) | NA | 40 (24) | 60 (35) | NA | NA |
| Mak *et al.* | NA | NA | NA | NA | NA | NA | NA | NA | NA |
| Bilaloglu *et al.* | NA | NA | NA | NA | NA | 451/829 (54) | 244/829 (29) | 146/829 (60)  **ALL thrombotic events** | 305/829 (52)  **ALL thrombotic events** |
| Longchanp *et al.* | NA | NA | NA | NA | 2/25 (8) | 5/25 (20) | 8/25 (32) | NA | NA |
| Bemtgen *et al.* | 28 days | NA | NA | NA | NA | 3/11 (27) | 7/11 (64) | NA | NA |
| Parzy et al. | NA | NA | NA | NA | NA | NA | 13/13 (100) | NA | NA |
| Yuriditsky *et al.* | 24 days | NA | NA | NA | “most” | 19/64 (30) | 20/64 (31) | NA | NA |
| Hékimian *et al.* | NA | NA | NA | NA | NA | NA | NA | 3/8 (38) | NA |

*Outcome results not provided in these papers for ICU subgroup

Abbreviations: CI = confidence interval, ICU = intensive care unit, IQR = inter-quartile range, LOS = length of stay, NA = not available, VTE = venous thrombotic events

eTable 2.

| **Section/topic** | **#** | **Checklist item** | **Reported on page #** |
| --- | --- | --- | --- |
| **TITLE** | | |  |
| Title | 1 | Identify the report as a systematic review, meta-analysis, or both. | 1 |
| **ABSTRACT** | | |  |
| Structured summary | 2 | Provide a structured summary including, as applicable: background; objectives; data sources; study eligibility criteria, participants, and interventions; study appraisal and synthesis methods; results; limitations; conclusions and implications of key findings; systematic review registration number. | 3 |
| **INTRODUCTION** | | |  |
| Rationale | 3 | Describe the rationale for the review in the context of what is already known. | 7 |
| Objectives | 4 | Provide an explicit statement of questions being addressed with reference to participants, interventions, comparisons, outcomes, and study design (PICOS). | 7 |
| **METHODS** | | |  |
| Protocol and registration | 5 | Indicate if a review protocol exists, if and where it can be accessed (e.g., Web address), and, if available, provide registration information including registration number. | 8 |
| Eligibility criteria | 6 | Specify study characteristics (e.g., PICOS, length of follow-up) and report characteristics (e.g., years considered, language, publication status) used as criteria for eligibility, giving rationale. | 8 |
| Information sources | 7 | Describe all information sources (e.g., databases with dates of coverage, contact with study authors to identify additional studies) in the search and date last searched. | 8 |
| Search | 8 | Present full electronic search strategy for at least one database, including any limits used, such that it could be repeated. | 41 |
| Study selection | 9 | State the process for selecting studies (i.e., screening, eligibility, included in systematic review, and, if applicable, included in the meta-analysis). | 8 |
| Data collection process | 10 | Describe method of data extraction from reports (e.g., piloted forms, independently, in duplicate) and any processes for obtaining and confirming data from investigators. | 9 |
| Data items | 11 | List and define all variables for which data were sought (e.g., PICOS, funding sources) and any assumptions and simplifications made. | 9 |
| Risk of bias in individual studies | 12 | Describe methods used for assessing risk of bias of individual studies (including specification of whether this was done at the study or outcome level), and how this information is to be used in any data synthesis. | 10 |
| Summary measures | 13 | State the principal summary measures (e.g., risk ratio, difference in means). | 10 |
| Synthesis of results | 14 | Describe the methods of handling data and combining results of studies, if done, including measures of consistency (e.g., I^2^) for each meta-analysis. | 10 |

| **Section/topic** | **#** | **Checklist item** | **Reported on page #** |
| --- | --- | --- | --- |
| Risk of bias across studies | 15 | Specify any assessment of risk of bias that may affect the cumulative evidence (e.g., publication bias, selective reporting within studies). | 10 |
| Additional analyses | 16 | Describe methods of additional analyses (e.g., sensitivity or subgroup analyses, meta-regression), if done, indicating which were pre-specified. | N/A |
| **RESULTS** | | |  |
| Study selection | 17 | Give numbers of studies screened, assessed for eligibility, and included in the review, with reasons for exclusions at each stage, ideally with a flow diagram. | Figure 1 |
| Study characteristics | 18 | For each study, present characteristics for which data were extracted (e.g., study size, PICOS, follow-up period) and provide the citations. | 29, 32 |
| Risk of bias within studies | 19 | Present data on risk of bias of each study and, if available, any outcome level assessment (see item 12). | 15, 42 |
| Results of individual studies | 20 | For all outcomes considered (benefits or harms), present, for each study: (a) simple summary data for each intervention group (b) effect estimates and confidence intervals, ideally with a forest plot. | 30, 34 |
| Synthesis of results | 21 | Present results of each meta-analysis done, including confidence intervals and measures of consistency. | 30, 32 |
| Risk of bias across studies | 22 | Present results of any assessment of risk of bias across studies (see Item 15). | 15, 42 |
| Additional analysis | 23 | Give results of additional analyses, if done (e.g., sensitivity or subgroup analyses, meta-regression [see Item 16]). | N/A |
| **DISCUSSION** | | |  |
| Summary of evidence | 24 | Summarize the main findings including the strength of evidence for each main outcome; consider their relevance to key groups (e.g., healthcare providers, users, and policy makers). | 13, 14, 15 |
| Limitations | 25 | Discuss limitations at study and outcome level (e.g., risk of bias), and at review-level (e.g., incomplete retrieval of identified research, reporting bias). | 18 |
| Conclusions | 26 | Provide a general interpretation of the results in the context of other evidence, and implications for future research. | 19 |
| **FUNDING** | | |  |
| Funding | 27 | Describe sources of funding for the systematic review and other support (e.g., supply of data); role of funders for the systematic review. | N/A |

eTable 3.

| **Search Number (#)** | **Name of Search** | **PubMed Search Code** | **Hits** |
| --- | --- | --- | --- |
| #1 | Date | (“2020/01/01”[Date – Publication] : “3000”[Date – Publication]) | 1,395,225 |
| #2 | COVID-19 | ((wuhan[All Fields] AND (“coronavirus”[MeSH Terms] OR “coronavirus”[All Fields])) AND 2019/12[PDAT] : 2030[PDAT]) OR 2019-nCoV[All Fields] OR 2019nCoV[All Fields] OR COVID-19[All Fields] OR SARS-CoV-2[All Fields] | 72,579 |
| #3 | Thrombosis | **embolism and thrombosis[MeSH Terms]** | 221,171 |
| #4 | Venous Thrombosis | ((((“venous thromboembolism”[MeSH Terms] OR (“venous”[All Fields] AND “thromboembolism”[All Fields])) OR “venous thromboembolism”[All Fields]) OR ((“venous thrombosis”[MeSH Terms] OR (“venous”[All Fields] AND “thrombosis”[All Fields])) OR “venous thrombosis”[All Fields])) OR ((“pulmonary embolism”[MeSH Terms] OR (“pulmonary”[All Fields] AND “embolism”[All Fields])) OR “pulmonary embolism”[All Fields])) OR ((((“venous thrombosis”[MeSH Terms] OR (“venous”[All Fields] AND “thrombosis”[All Fields])) OR “venous thrombosis”[All Fields]) OR (“vein”[All Fields] AND “thrombosis”[All Fields])) OR “vein thrombosis”[All Fields]) | 151,039 |
| #5 | Arterial Thrombosis, Stroke, Myocardial infarction, Mesenteric ischaemia | ((((“myocardial infarction”[MeSH Terms] OR (“myocardial”[All Fields] AND “infarction”[All Fields])) OR “myocardial infarction”[All Fields]) OR (((“stroke”[MeSH Terms] OR “stroke”[All Fields]) OR “strokes”[All Fields]) OR “stroke s”[All Fields])) OR ((((“mesenteric ischemia”[MeSH Terms] OR (“mesenteric”[All Fields] AND “ischemia”[All Fields])) OR “mesenteric ischemia”[All Fields]) OR (“mesenteric”[All Fields] AND “ischaemia”[All Fields])) OR “mesenteric ischaemia”[All Fields])) OR ((((((((((((((((“arterialization”[All Fields] OR “arterializations”[All Fields]) OR “arterialize”[All Fields]) OR “arterialized”[All Fields]) OR “arterializing”[All Fields]) OR “arterially”[All Fields]) OR “arterials”[All Fields]) OR “arterie”[All Fields]) OR “arteries”[MeSH Terms]) OR “arteries”[All Fields]) OR “arterial”[All Fields]) OR “arteris”[All Fields]) OR “artery”[All Fields]) OR “arterious”[All Fields]) OR “artery s”[All Fields]) OR “arterys”[All Fields]) AND (((((“thrombose”[All Fields] OR “thrombosing”[All Fields]) OR “thrombosis”[MeSH Terms]) OR “thrombosis”[All Fields]) OR “thrombosed”[All Fields]) OR “thromboses”[All Fields])) | 630,181 |
| #6 |  | #3 OR #4 OR #5 | 816,799 |
| #7 |  | #1 AND #2 AND #6 | 2,429 |
|  |  | **TOTAL RECORDS IDENTIFIED** | **2,429** |

eTable 4.

| **Study** | **Inclusion criteria** | **Interventions** | **Diagnosis** | **Overall risk of bias** |
| --- | --- | --- | --- | --- |
| Cui *et al.* | - Retrospective - Single-centre - Small: 81 - All patients in ITU with severe infection | - Not clear if all had routine screening for thrombotic events - None had thromboprophylaxis | - **DVT** - Not reporting other events | **High**   - Small - Retrospective - Routine screening not clear - Not reporting all events - 11% remain in hospital |
| Helms *et al.* | - Prospective - Multi-centre - Large: 150 - All patients in ITU with severe infection | - No routine screening for thrombotic events - 100% had at least thromboprophylaxis | - **DVT** - **PE** - RRT **circuit thrombosis** - ECMO **pump thrombosis** - **Arterial** thrombotic events | **Low**   - 67% remain in hospital and therefore at risk of further events |
| Klok *et al.* | - Retrospective - Multi-centre - Large: 184 - All patients in ITU with severe infection | - No routine screening for thrombotic events - 100% had at least thromboprophylaxis | - **DVT** - **PE** - **Arterial** thrombotic events | **Low**   - 35% of patients still remain in hospital |
| Poissy *et al.* | - Retrospective - Single centre - Large: 107 - All patients in ITU with severe infection | - No routine screening for thrombotic events | - **PE** - **DVT** | **Moderate**   - Not reporting arterial events - Baseline characteristics of whole cohort not included - 41% still remain in hospital |
| Thomas *et al.* | - Retrospective - Single centre - Small: 63 - All patients in ITU with severe infection | - No routine screening for thrombotic events. - 100% had at least thromboprophylaxis | - **PE** - **DVT** - **Line thrombosis** - **Arterial** thrombotic events - **Not reporting circuit thrombosis** | **Moderate**   - Small - Retrospective - 44% remain in ITU - Not including patients on ECMO – transferred to tertiary centre |
| Lodigiani *et al.* | - Retrospective - Single centre - Large: 388 (61 admitted to ITU) | - No routine screening for thrombotic events - 100% ITU patients had at least thromboprophylaxis | - **PE** - **DVT** - **Arterial** thrombotic events | **Moderate**   - Retrospective - Small |
| Beyls *et al.* | - Retrospective - Single centre - Small: 12 patients - Only including patients on ECMO | - No routine screening for thrombotic events - Heparin on ECMO | - **PE** - **Cannula thrombosis** - **DVT** - **Oxygenator thrombosis** - Not reporting arterial thrombotic events | **Critical**   - Only including those patients on ECMO - Small - Single centre - 83% of patients remain in hospital |
| Maatman *et al.* | - Retrospective - Multicentre - Large: 109 - All patients in ITU with severe infection | - No routine screening for thrombotic events - 100% had thromboprophylaxis | - **PE** - **DVT** - **Line thrombosis** - **Not** reporting **RRT circuit thrombosis.** - **Not** reporting **arterial** thrombotic events | **Moderate**   - Not reporting arterial events |
| Llitjos *et al.* | - Retrospective - Multi-centre - Small: 26 - All patients in ITU with severe infection | - Routine complete duplex ultrasound. Second scan if first negative at 7 days. - 100% had at least thromboprophylaxis | - **PE** - **DVT** - **Not** reporting **arterial** thrombotic events | **Moderate**   - Small - Retrospective - Not reporting all venous and arterial events. - All patients had routine screening with ultrasound – giving weighting to DVT |
| Ren *et al.* | - Prospective - Multi-centre - Small: 48 - All patients in ITU with severe infection | - Routine complete duplex ultrasound - 100% had at least thromboprophylaxis unless contraindicated | - **DVT** - **Not** reporting other **venous nor arterial** thrombotic events. | **High**   - Small - Only reporting DVT - F/U time and remining patients at risk of further events not known. - All patients had routine screening with ultrasound – giving weighting to DVT |
| Nahum *et al.* | - Prospective - Single centre - Small: 34 - All patients in ITU with severe infection | - Routine complete duplex ultrasound. Second scan after 48 hours if first scan negative - 100% had thromboprophylaxis | - **DVT** - **Not** reporting other **venous and arterial** thrombotic events | **High**   - Small - Only reporting DVT - F/U time and remining patients at risk of further events not known. - All patients had routine screening with ultrasound – giving weighting to DVT |
| Fraisse *et al.* | - Retrospective - Single centre - Small: 92 - Excluded RRT filter and CVC thrombosis | - No routine screening for thrombotic events - 100% had at least thromboprophylaxis | - **PE** - **DVT** - **Arterial thrombotic events** | **Low**   - Small - All events reported, but did exclude RRT and CVC thrombosis. - 27% remain on ITU. |
| Al Samkari *et al.* | - Retrospective - Multicentre - Large: 144 (ICU) - Those who had d-dimer measured - Critical illness defined as need for intubation. (Different from above studies) | - No routine screening for thrombotic events - 100% had at least thromboprophylaxis | - **PE** - **DVT** - **Other venous and arterial thrombotic events** | **Low**   - Large - All events reported. - However, definition of severe Illness differs from that of other studies – all patients in this study were intubated. |
| Hippensteel *at al.* | - Retrospective - Single centre - Small: 91 - Excluded patients if on therapeutic anticoagulation. - Excluded ECMO patients. | - No routine screening for thrombotic events - Thromboprophylaxis not reported | - **PE** - **DVT** - **Not** reporting other **venous and arterial** thrombotic events | **High**   - Excluded ECMO patients. - Not reporting all venous and arterial events. - Thromboprophylaxis not reported. - 28% remain in hospital |
| Desborough *et al.* | - Retrospective - Single centre - Small: 66 - All patients in ITU with severe infection | - No routine screening for thrombotic events - 100% had at least thromboprophylaxis | - **PE** - **DVT** - **Not** reporting other **venous and arterial** thrombotic events | **Moderate**   - Small - Retrospective - Not reporting all venous and arterial events. - 12% remain in hospital |
| Criel *et al.* | - Prospective - Single centre - Small: 30 - All patients in ITU with severe infection | - Routine complete duplex ultrasound - 100% had at least thromboprophylaxis | - **DVT** - **Not** reporting other **venous and arterial** thrombotic events | **High**   - Small - Only reporting DVT - Number remaining in hospital not known. - All patients had routine screening with ultrasound – giving weighting to DVT |
| Middeldorp *et al.* | - Retrospective - Single centre - Large: 198 (75 admitted to ITU) - All patients on ITU with severe infection | - Bilateral leg ultrasound screening performed in 51% of patients - Thromboprophylaxis rate in ICU group not reported | - **PE** - **DVT** - **Not** reporting other **venous and arterial** thrombotic events | **Moderate**   - Retrospective - Not reporting all venous and arterial events - 51% of patients had routine screening with ultrasound – giving weighting to DVT |
| Voicu *et al.* | - Prospective - Single centre - Small: 92 - All patients in ITU with severe infection | - Routine complete duplex ultrasound. Second scan if first negative at 7 days. - 100% had at least thromboprophylaxis | - **PE** - **DVT** - **Not** reporting other **venous** **and arterial** thrombotic events | **High**   - Small - Not reporting all venous and arterial events - Duration of follow-up, and total number of patients still in hospital at time of analysis not clear. - All patients had routine screening with ultrasound – giving weighting to DVT |
| Longhitano *et al.* | - Retrospective - Single centre - Small: 62 - All patients in ITU with severe infection | - No routine screening for thrombotic events - 100% had thromboprophylaxis | - **PE** - **DVT** - **Not** reporting other **venous and arterial** thrombotic events | **High**   - Retrospective - Small - Not reporting all venous and arterial events - Duration of follow-up, and total number of patients still in hospital at time of analysis not clear. - Some patients managed at another site – captured all events? |
| Shah *et al.* | - Retrospective - Multicentre - Large: 187 - All patients in ITU with severe infection | - No routine screening for thrombotic events - 81% had thromboprophylaxis - 14% had therapeutic anticoagulation | - **PE** - **DVT** - **Other venous and arterial thrombotic events** | **Low**   - Large - Multicentre - Reporting all thrombotic events - No routine screening. |
| Piazza *et al.* | - Retrospective - Multicentre - Large: 170 - All patients with the infection. Subgroup analysis in ITU patients | - No routine screening for thrombotic events - 89% had thromboprophylaxis - Unclear how many patients had therapeutic anticoagulation. | - **PE** - **DVT** - **Arterial thrombotic events** | **Low**   - Large - Multicentre - Reporting all events - 29% still hospitalised at time of analysis |
| Mak *et al.* | - Retrospective - Single centre - Small: 51 patients - All ECMO admissions | - Routine screening for PE with CTPA - Unclear how many patients had thromboprophylaxis | - **PE** - **Not** reporting rate of **DVT** - **Not** reporting other **venous and arterial** thrombotic events | **Critical**   - Small - Retrospective - Single centre - Including only ECMO patients - Only reporting PE - All patients underwent systematic screening with CTPA - Unknown thromboprophylaxis strategy |
| Bilaloglu *et al.* | - Retrospective - Multicentre - Large: 829 - All patients with the infection. Subgroup analysis in ITU patients | - No routine screening for thrombotic events - Unclear how many patients had thromboprophylaxis | - **PE** - **DVT** - **Arterial thrombotic events** | **Low**   - Large - Multicentre - Reporting all events - However, actual thromboprophylaxis rate unknown |
| Longchamp *et al.* | - Retrospective - Single centre - Small: 25 patients | - Routine screening with lower limb ultrasound doppler (between day 5 and 10) - 96% had thromboprophylaxis | - **PE** - **DVT** - **Not** reporting other **venous and arterial** thrombotic events | **High**   - Small - Single centre - Retrospective - Not reporting all events. - All patients had routine screening with ultrasound – giving weighting to DVT |
| Bemtgen *et al.* | - Retrospective - Single centre - Small: 11 patients - Only including patients on ECMO | - No routine screening for thrombotic events - UFH for all patients on ECMO | - **ECMO thrombotic events only** | **Critical**   - Small - Retrospective - Only reporting ECMO thrombotic events - Including only ECMO patients |
| Parzy *et al* | - Retrospective - Single centre - Small: 13 patients - Only including patients on ECMO | - Routine screening with contrast CT following ECMO retrieval and weaning. - UFH for all patients on ECMO | - **PE** - **DVT** - **ECMO thromboses** | **High**   - Small - Single centre - Retrospective - All patients had routine screening with CT |
| Yuriditsky *et al.* | - Retrospective - Single centre - Small: 64 patients - All patients in ITU with severe infection | - No routine screening - 14% had thromboprophylaxis - 86% had therapeutic anticoagulation | - **PE** - **DVT** - **Not** reporting other **venous and arterial** thrombotic events | **Moderate**   - Small - Single centre - Retrospective - Not reporting all events |
| Hekimian *et al.* | - Retrospective - Single centre - Small: 51 patients - All patients in ITU with severe infection | - No routine screening - Thromboprophylaxis unclear | - **Massive PE only** - **Not** reporting **DVT** or other **venous and arterial** thrombotic events | **High**   - Small - Single centre - Retrospective - Only reporting massive PE - Remaining patients at risk at the time of analysis unknown. |

Abbreviations: CTPA = computed tomography pulmonary angiogram, CVC = central venous catheter, DVT = deep vein thrombosis, ECMO = extracorporeal membrane oxygenator, ITU = intensive treatment unit, PE = pulmonary embolus, RRT = renal replacement therapy, UFH = unfractionated heparin
